# Prevalence, hematological parameters, and coagulation profiles: Cardiovascular diseases statistics in the Asir region, Saudi Arabia

**DOI:** 10.15537/smj.2023.44.4.20220746

**Published:** 2023-04

**Authors:** Kholoud N. Almetairi, Sultan Z. Alasmari, Mohammed H. Makkawi, Ahmad A. Shaikh

**Affiliations:** *From the Department of Clinical Laboratory Sciences (Kholoud, Sultan, Mohammed, Ahmed), Faculty of Applied Medical Sciences, King Khalid University, Abha; from Department of Laboratory (Kholoud), Prince Faisal bin Khalid Cardiac Center, Abha, Kingdom of Saudi Arabia.*

**Keywords:** prevalence, CVD, hematological parameters, coagulation profiles, inflammation

## Abstract

**Objectives:**

To determine the prevalence of cardiovascular disease (CVD) types in the Asir region of Saudi Arabia and the importance of hematological testing for CVD patients in the context of disease management.

**Methods:**

This retrospective study comprised 416 CVD patients, and samples were divided based the type of CVD. The Mann Whitney U test was used to compare patients’ hematological markers and coagulation profiles to those of healthy controls.

**Results:**

The rate of ischemic heart disease (IHD) was 80.7% that of other CVDs, and the rate of ST-elevation myocardial infarction (STEMI) was 37.3% the rate of CVD. Significant differences were observed in the hematological and coagulation parameters of CVD patients compared to the control group. White blood cells (WBC) were significantly higher in STEMI, non-ST-elevation myocardial infarction (NSTEMI), unstable angina (UA), and heart failure (HF) groups. Red blood cells (RBC) were significantly lower in STEMI, NSTEMI, UA, chronic coronary syndrome (CCS), HF, dilated cardiomyopathy (DCM), and ischemic cardiomyopathy (ICM). Red distribution width (RDW) was significantly greater in the HF, DCM, and ICM groups. Prothrombin time (PT) was significantly higher in the STEMI, HF, and DCM groups.

**Conclusion:**

ST-elevation myocardial infarction has a higher prevalence rate among CVD patients in the Asir region. Both coagulation and hematological indicators have high potential utility as CVD diagnostic and prognostic markers.

**C**ardiovascular disease (CVD) is one of the leading causes of mortality and morbidity. As a term, CVD refers to a wide range of conditions that affect the heart and blood vessels; it thereby encompasses heart failure (HF), atherosclerosis, myocardial infarction (MI), hypertension, coronary artery disease (CAD), coronary heart disease (CHD), and acute coronary syndrome (ACS).^1,2^ Despite recent advances in medication and technology, CVD mortality and morbidity remain high,^2^ with 17.3 million deaths attributed worldwide according to the 2013 Global Burden of Disease (GBD) study; moreover, CVD caused more deaths than all communicable, maternal, neonatal, and nutritional illnesses combined, accounting for 31.5% of all deaths and 45% of non-communicable disease (NCD) deaths.^3^ According to a prediction of the World Health Organization (WHO), the number of individuals suffering from CVD is expected to exceed 23.6 million by 2030.^4^

In Saudi Arabia, NCDs overall account for approximately 73% of all mortality and CVD in particular is the main cause of death, accounting for approximately 37% of all NCD deaths.^5,6^ Ischemic heart disease (IHD) and stroke are among the top 10 causes of death in the Middle East, with IHD ranking second and stroke ranking fourth in Saudi Arabia.^7^ Several risk factors contribute to the development of CVD, a few of which are hypertension (HTN), diabetes mellitus (DM), hypercholesterolemia (HyCh), cigarette smoking, and obesity. Controlling these factors may contribute to the management and prevention of CVD occurrences.

When CVD occurs, early and accurate detection is essential for disease control and management. Diagnosis of CVDs generally requires clinical evaluation utilizing costly procedures; however, physicians frequently use the complete blood count (CBC) and other routine tests to monitor the health of both sick and healthy people. The low cost and accessibility of these tests make them ideal for investigating and diagnosing concerns such as anemia, infection risk, hematologic malignancies, and coagulation abnormalities.^8^

The hematological parameters of CVD patients show functional disturbances owing to the involvement of inflammation and systemic hypoxemia in the pathophysiological processes. Several studies have identified a link between risk of adverse outcomes in CVD patients and hematological parameters which could lead to circulatory failure; thus, hematological and coagulation markers have prognostic significance in CVD.^9^

In patients with chronic heart failure (CHF), increased red distribution width (RDW) is a significant independent predictor of increased morbidity and mortality.^10^ Red distribution width has also been associated with inflammation in rheumatoid arthritis, cancer, autoimmune hepatitis, COVID-19 infection, and autoimmune diseases.^11^ Mean platelet volume (MPV) is a platelet activation measure that is commonly utilized for routine screening and has been demonstrated to be a prognostic predictor in CVD patients.^12^ The MPV hemogram measurement has been associated with a diverse range of inflammatory diseases, including type 2 DM, diabetic nephropathy, hypothyroidism, vertebral discopathies, infection, irritable bowel syndrome, rheumatoid arthritis, obesity, mortality in ICU population, and liver fibrosis.^13-22^ As inflammation and cardiovascular conditions are closely related, RDW, MPV, and other hematological markers investigations in cardiac conditions are of interest. There is additional evidence that RDW, white blood cells (WBC), and MPV, when combined with the coagulation profile, can contribute to the diagnosis of acute coronary syndrome (ACS) in individuals presenting with chest pain.^23^ Thus, CBC can be utilized to monitor CVDs and evaluate patient prognosis.^8^ However, the importance of these hematological markers in predicting in-hospital and short-term outcomes in patients with ACS and other CVDs remains to be determined.^24^

This study aims to assess the prevalence of various types of CVD in the Asir region, Saudi Arabia, as well as the relevance of hematological and coagulation biomarkers for CVD patients in the context of subsequent management and care.

## Methods

This retrospective study was carried out by collecting patient data of hematological parameters and coagulation profiles from Prince Faisal bin Khalid Cardiac Center, Abha, Asir region, Saudi Arabia. Patients with CVD who were admitted to the hospital as inpatients between December 2020 and January 2022 were included in the study. A total of 54 healthy individuals without health issues were collected from the blood bank unit and screened for comparison with the patients’ samples.

The diagnosis, age, gender, and medical history of CVD patients were provided by the medical record department, while hematological and coagulation parameters were provided by the laboratory department for each patient. Patients were classified into categories according to the type of cardiovascular disease they suffered. Patients with a history of CVD met the inclusion criteria. Patients who were anemic or who had an infection were excluded. Ethical approval (Approval No: 2022-2119) for this study was obtained from the research ethics committee at King Khalid University (HAPO-06-B-001).

### Statistical analysis

The GraphPad Prism (version 9.3.1.471) and Excel (version 22052000) were used. The quantitative variables (age, WBC, red blood cells [RBC], hemoglobin [HGB], hematocrit [HCT], mean corpuscular volume [MCV], mean corpuscular hemoglobin [MCH], mean corpuscular hemoglobin concentration [MCHC], Red distribution width [RDW], platelet count [PLT], MPV, prothrombin time (PT), partial thromboplastin time [PTT], and international normalized ratio [INR]) were presented as mean and standard error of the mean (SEM), whereas the qualitative variables (gender, nationality, prior history, diagnosis) were presented as frequency and percentage. To compare study parameters between CVD and control groups, the nonparametric Mann-Whitney U test was performed. Kalmogorove Smrinov (K-S) test was used to check the data normality. Statistical significance was defined as a *p*-value of <0.05.

## Results

**Figure 1:**
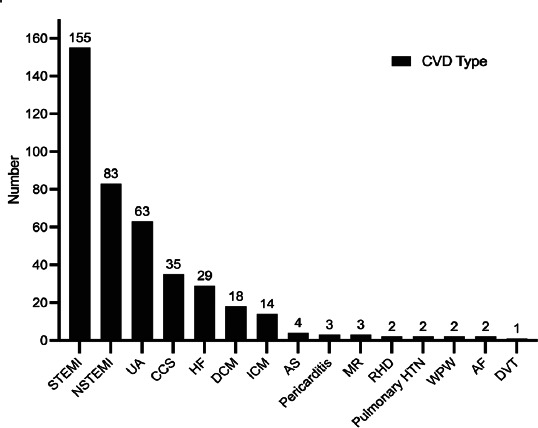
- The prevalence of cardiovascular diseases (CVD) in Asir region. Types were listed in descending order from most common to least (left to right). STEMI: ST-elevation myocardial infarction, NSTEMI: non-ST-elevation myocardial infarction, UA: unstable angina, CCS: chronic coronary syndrome, HF: heart failure, DCM: dilated cardiomyopathy, ICM: ischemic cardiomyopathy, AS: Aortic stenosis, MR: mitral regurgitation, RHD: rheumatic heart disease, HTN: hypertension, WPW: Wolff-Parkinson-White syndrome, AF: atrial fibrillation, DVT: deep vein thrombosis

A total of 433 patients with CVD were investigated, 416 patients fulfilling the criteria and the remainder being ruled out. A total of 416 CVD patients with an average age of 60 years were included in the study (79.3% men and 20.7% women). Saudi citizens comprise most of the patients. ST-Elevation Myocardial Infarction (STEMI), as shown in Figure 1, was the most prevalent forms of CVD. The clinical and paraclinical characteristics of CVD patients are represented in Table 1.

**Table 1:** - Cardiovascular disease (CVD) patients’ clinical/paraclinical data (N=416).

Characteristics	n	(%)
Age (Years)	(17-109)	
***Gender***		
Male	330	79.3
Female	86	20.7
***Nationality***		
Saudi	344	82.7
Non-Saudi	72	17.3
***Previous history***		
Smoking	65	15.6
Diabetes mellitus	216	51.8
Hypertension	237	56.9
***Diagnosis***		
IHD (STEMI)	155	37.3
IHD (NSTEMI)	83	20.2
IHD (unstable angina)	63	15.0
IHD (CCS)	35	8.4
Heart failure	29	7.0
DCM	18	4.3
ICM	14	3.4
Aortic stenosis	4	1.0
Pericarditis	3	0.7
Mitral regurgitation	3	0.7
RHD	2	0.5
Pulmonary hypertension	2	0.5
WPW	2	0.5
Atrial fibrillation	2	0.5
DVT	1	0.2

Values are presented as number and percentages (%). IHD: ischemic heart disease, STEMI: ST-elevation myocardial infarction, NSTEMI: non-ST-elevation myocardial infarction, DCM: dilated cardiomyopathy, ICM: ischemic cardiomyopathy, CCS: chronic coronary syndrome, RHD: rheumatic heart disease DVT: deep vein thrombosis, WPW: Wolff-Parkinson-White syndrome

**Figure 2:**
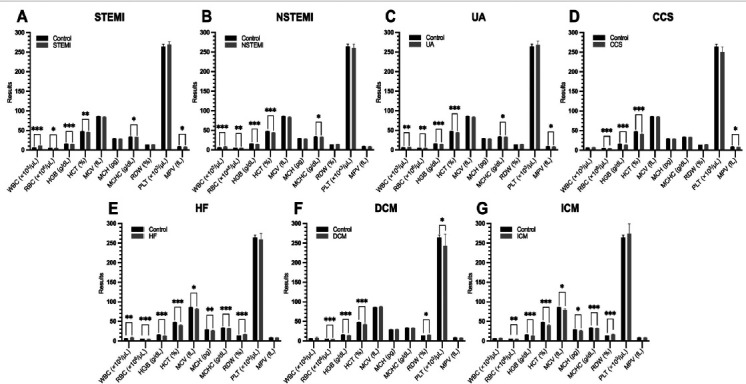
- Comparison of hematological parameters between ST-elevation myocardial infarction (STEMI), non-ST-elevation myocardial infarction (NSTEMI), unstable angina (UA), chronic coronary syndrome (CCS), heart failure (HF), dilated cardiomyopathy (DCM), and ischemic cardiomyopathy (ICM) patients with control group. The total sample consisted of 397 cardiovascular disease patients and 54 controls. *p<0.05, **p<0.01, ***p<0.001. WBC: white blood cells, RBC: red blood cells, HGB: hemoglobin, HCT: hematocrit, MCV: mean corpuscular volume, MCH: mean corpuscular hemoglobin, MCHC: mean corpuscular hemoglobin concentration, RDW: Red distribution width, PLT: count, MPV: mean platelet volume

To determine the values of hematological parameters of the most common CVD in the center, patients’ results were extracted and analyzed. Data demonstrated that the STEMI group had considerably higher WBC (median=10.71, SEM=0.30) with *p*<0.001 and significantly lower RBC (median=5.41, SEM=0.06) with *p*=0.01, HGB (median=15.51, SEM=0.16) with *p*<0.001, HCT (median=45.83, SEM=0.45) with *p*=0.001, MCHC (median=33.79, SEM=0.10) with *p*=0.02, and MPV (median=8.46, SEM=0.09) with *p*=0.010 as compared to the control group (Figure 2A). Non-ST-elevation myocardial infarction (NSTEMI) group had considerably greater WBC (median=8.1, SEM=0.33) with *p*<0.001 and a significant reduced RBC (median=5.25, SEM=0.09) with *p*=0.002, HGB (median=14.9, SEM=0.23) with *p*<0.001, HCT (median=43.87, SEM=0.66) with *p*<0.001, and MCHC (median=33.7, SEM=0.14) with *p*=0.010 as compared to the control group (Figure 2B). Data demonstrated that the UA group had considerably greater WBC (median=7.62, SEM=0.29) with *p*=0.006 and significantly reduced RBC (median=5.31, SEM=0.09) with *p*=0.001, HGB (median=15.01, SEM=0.24) with *p*<0.001, HCT (median=44.09, SEM=0.68) with *p*<0.001, MCHC (median=33.8, SEM=0.14) with *p*=0.02 and MPV (median=8.48, SEM=0.14) with *p*=0.01 as compared to control group (Figure 2C). Chronic coronary syndrome (CCS) group had considerably a reduction of RBC (median=5.001, SEM=0.11) with *p*<0.001, HGB (median=14.18, SEM=0.37) with *p*<0.001, HCT (median=42.18, SEM=0.95) with *p*<0.001 and MPV (median=8.39, SEM=0.14) with *p*=0.01 as compared to the control group (Figure 2D). The HF group had considerably greater WBC (median=7.65, SEM=0.72) with *p*=0.002, RDW (median=15.91, SEM=0.64) with *p*<0.001 and significantly reduced RBC (median=5.04, SEM=0.16) with *p*<0.001, HGB (median=13.46, SEM=0.55) with *p*<0.001, HCT (median=41.11, SEM=1.52) with *p*<0.001, MCV (median=82.0, SEM= 1.76) with *p*=0.046, MCH (median=27.69, SEM=0.78) with *p*=0.003 and MCHC (median=32.7, SEM=0.34) with *p*<0.001 as compared to the control group (Figure 2E). Data demonstrated that the dilated cardiomyopathy (DCM) group had considerably greater RDW (median=15.0, SEM=0.6) with *p*=0.03 and significantly reduced RBC (median=4.92, SEM=0.16) with *p*<0.001, HGB (median=14.67, SEM=0.46) with *p*<0.001, HCT (median=43.50, SEM=1.15) with *p*<0.001, and PLT (median=207.25, SEM=30.27) with *p*=0.01 as compared to the control group (Figure 2F). Ischemic cardiomyopathy group had considerably greater RDW (median=15.84, SEM=0.87) with *p*<0.001 and significantly reduced RBC (median=5.09, SEM=0.21) with *p*=0.009, HGB (median=13.19, SEM=0.68) with *p*<0.001, HCT (median=39.93, SEM=1.82) with *p*<0.001, MCV (median=79.2, SEM=3.25) with *p*=0.04, MCH (median=26.17, SEM=1.24) with *p*=0.02 and MCHC (median=33.05, SEM=0.33) with *p*<0.001 as compared to control group (Figure 2G).

**Figure 3:**
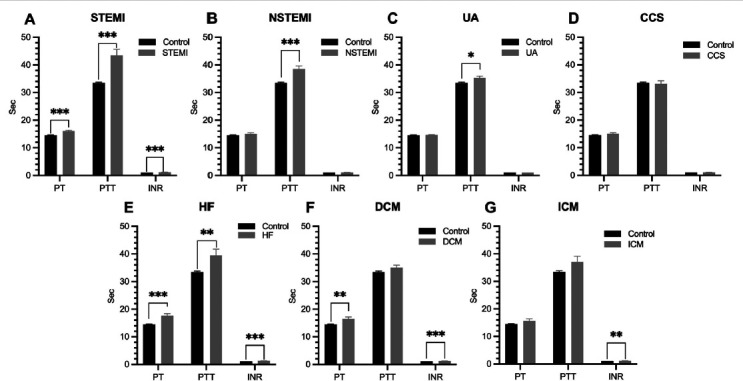
- Comparison of coagulation profiles of ST-elevation myocardial infarction (STEMI), non-ST-elevation myocardial infarction (NSTEMI), unstable angina (UA), chronic coronary syndrome (CCS), heart failure (HF), dilated cardiomyopathy (DCM), and ischemic cardiomyopathy (ICM) patients with control group. The total sample consisted of 397 cardiovascular disease patients and 54 controls. *p<0.05, **p<0.01, ***p<0.001. PT: prothrombin time, PTT: partial thromboplastin time, INR: international normalized ratio

To determine the values of coagulation profiles of the most common cardiovascular diseases in the center, patients’ results were extracted and analyzed. Data of STEMI patients showed that PT (median=15.4, SEM=0.28) with *p*<0.001, PTT (median=37.6, SEM=2.16) with *p*<0.001, and INR (median=1.12, SEM=0.02) with *p*<0.001 are substantially higher in the STEMI group compared to the control group (Figure 3A). While data determined that PTT (median=36.7, SEM=1.08) with *p*<0.001 are substantially greater in the NSTEMI group compared to the control group (Figure 3B). Unstable angina group had considerably PTT (median=35.3, SEM=0.59) with *p*=0.02 greater than the control group (Figure 3C). But no statistically significant observation was shown in the CCS group compared to the control group (Figure 3D). Data determined that PT (median=17.1, SEM=0.77) with *p*<0.001, PTT (median=36.0, SEM=2.28) with *p*=0.001 and INR (median=1.26, SEM=0.06) with *p*<0.001 are substantially greater in the HF group compared to control group (Figure 3E). Research observation determined that PT (median=16.8, SEM=0.69) with *p*=0.002 and INR (median=1.23, SEM=0.05) with *p*<0.001 are substantially greater in DCM group compared to control group (Figure 3F). While INR only (median=1.135, SEM=0.05) with *p*=0.006 are substantially greater in the ischemic cardiomyopathy (ICM) group compared to control group (Figure 3G). However, the *p*-value of PT =0.05, which is approaching the significance threshold.

The values of hematological parameters and coagulation profiles among less prevalent CVD patients were examined and determined to assess the relevance of such values for disease prognosis and prevention. Tables 2 & 3 demonstrated data of hematological parameters and coagulation profiles of less prevalent diseases that include aortic stenosis (AS), pericarditis, mitral regurgitation (MR), rheumatic heart disease (RHD), pulmonary hypertension (HTN), Wolff-Parkinson-White syndrome (WPW), atrial fibrillation (AF), and deep vein thrombosis (DVT).

**Table 2:** - The hematological parameters of less prevalent cardiovascular disease (CVD) groups.

Hematological parameters	AS	Pericarditis	MR	RHD	Pulmonary HTN	WPW	AF	DVT
	Median, *p*-value							
WBC (×103/µL)	6.19, (0.84)	8.19, (0.08)	6.67, (>0.99)	7.72, (0.52)	6.02, (0.81)	9.93, (0.03*)	8.6, (0.19)	8.45
RBC (×10^6^/µL)	4.75, (0.001)*	5.79, (0.79)	5.19, (0.15)	4.81, (0.03)*	5.35, (0.97)	5.24, (0.14)	5.04, (0.05)	4.64
HBG (g/dL)	14.8, (0.01)*	16.15, (0.77)	14.99, (0.03)*	14.7, (0.05)	15.5, (0.91)	15.36, (0.57)	14.47, (0.04)*	12.85
HCT (%)	43.3, (0.002)*	47.19, (0.77)	43.7, (0.01)*	43.2, (0.03)*	46.4, (>0.99)	44.95, (0.34)	43.4, (0.04)*	38.5
MCV (fL)	88.65, (0.08)	88.53, (0.82)	76.6, (0.12)	89.8, (0.13)	86.6, (0.62)	85.7, (0.92)	86.28, (0.99)	83.03
MCH (pg)	30.45, (0.048)*	29.87, (0.69)	26.3, (0.21)	30.6, (0.25)	28.99, (0.81)	29.3, (>0.99)	28.75, (0.77)	27.72
MCHC (g/dL)	34.63, (0.23)	34.23, (0.52)	34.3, (0.9)	34.1, (0.89)	33.43, (0.18)	34.1, (0.94)	33.29, (0.13)	33.39
RDW (%)	14.23, (0.71)	13.7, (0.48)	15.5, (0.36)	13.4, (0.51)	16.9, (0.22)	15.75, (0.9)	13.49, (0.2)	14.69
PLT (×10^3^/µL)	193.65, (0.32)	244.8, (0.67)	266.4, (0.9)	242.1, (0.68)	263.5, (0.91)	190.9, (0.01)*	285.2, (0.57)	245.4
MPV (fL)	8.65, (0.60)	7.54, (0.01)*	8.68, (0.44)	9.56, (0.71)	7.54, (0.05)	10.6, (0.03)*	8.21, (0.17)	9.86

*Statistically significant. WBC: white blood cells, RBC: red blood cells, HGB: hemoglobin, HCT: hematocrit, MCV: mean corpuscular volume, MCH: mean corpuscular hemoglobin, MCHC: mean corpuscular hemoglobin concentration, RDW: Red distribution width, PLT: count, MPV: mean platelet volume, AS: Aortic stenosis, MR: mitral regurgitation, RHD: rheumatic heart disease, HTN: hypertension, WPW: Wolff-Parkinson-White syndrome, AF: atrial fibrillation, DVT: deep vein thrombosis

**Table 3:** - The coagulation profiles of less prevalent cardiovascular disease groups.

Coagulation parameters	AS	Pericarditis	MR	RHD	Pulmonary HTN	WPW	AF	DVT
	Median, *p*-value								
PT (Sec)	15.85, (0.009)*	14.9, (0.4)	17.1, (0.34)	15.35, (0.07)	15.05, (0.25)	14.35, (0.91)	15.25, (0.21)	14.5
PTT (Sec)	31.55, (0.56)	37.4, (0.02)*	36.3, (0.11)	31.95, (0.36)	38.3, (0.02)*	29.9, (0.04)*	34.1, (0.94)	54
INR (Sec)	1.55, (0.01)*	1.08, (0.4)	1.26, (0.01)*	1.12, (0.06)	1.095, (0.27)	1.045, (0.95)	1.11, (0.22)	1.05

PT: prothrombin time, PTT: partial thromboplastin time, INR: international normalized ratio, AS: Aortic stenosis, MR: mitral regurgitation, RHD: rheumatic heart disease, HTN: hypertension, WPW: Wolff-Parkinson-White syndrome, AF: atrial fibrillation, DVT: deep vein thrombosis

## Discussion

In Saudi Arabia, CVD is one of the primary causes of death.^4^ Cardiovascular disease causes a wide range of impairments to the body, involving variations in hematological parameters. The inflammatory and hypoxemic nature of CVD activates bone marrow, resulting in the release of immature cells or an increase in other cells in circulation, depending on the degree of stimulation.^9^ The current study investigated the prevalence of numerous forms of CVD in the Asir region, as well as how valuable hematological and coagulation biomarkers are for CVD patients on the basis of further control and management.

The study found that the risk of IHD was 80.7% higher in CVD than in other types. Moreover, STEMI has a greater rate at 37.3% than other types of CVD. Ischemic heart disease is well addressed in the literature as being the major cause of death among CVD patients.^25^ Several prior studies revealed that hematological or coagulation parameters had a role in the diagnosis and prognosis of CVD.^23,26^ In line with previous studies, there had been a variance in the hematological and coagulation parameters of different types of CVD, as well as a variable in the prevalence of CVD by gender.

Ischemic heart disease affects roughly 126 million individuals (1,655 per 100,000), accounting for 1.72% of the global population. It has caused the deaths of 9 million people worldwide. The global incidence of IHD is rising, and by 2030, it will have exceeded 1,845 million people.^27^ According to our findings, overall IHD (80.7%) had the greatest incidence rate in the Asir region, which is consistent with prior research.

Our data show that men had a higher prevalence of CVD than women, which is consistent with prior research.^27,28^ Women, on the other hand, have a higher death rate and a worse prognosis following an acute cardiovascular (CV) event than males.^28^ The cause of such gender differences would be estrogen’s protective effect on the development of CVD risk factors, particularly hypertension and dyslipidemia. Premenopausal women exhibited lower systolic blood pressure, higher high-density lipoprotein (HDL) cholesterol, and lower triglyceride levels than males, presumably due to estrogen. Even though the exact mechanism of this protection is unknown, estrogen is known to have a variety of effects on the atherosclerotic and blood-lipid management processes. The fact that this protection is lost following menopause contributes to the evidence that estrogen has a protective effect. Women, on the other hand, had greater rates of hypertension and cardiovascular illness by the age of 75 than males.^29^

An increase in total WBC is a typical laboratory result during inflammation. Cardiovascular diseases have been attributed to inflammation and inflammatory markers, and it has been demonstrated that atherosclerosis has a key inflammatory basis as a multifactorial disease that predisposes to the majority of CVDs, including myocardial infarction (MI) and heart failure (HF). Despite the fact that the association between inflammation and CVDs is not entirely understood, examining inflammatory markers is expected to be beneficial in determining the prognosis of CVD patients.^8^ Consequently, our study indicated that WBC levels were considerably higher in the STEMI, NSTEMI, UA, and HF groups, as comparable to findings obtained in other investigations.^24,30^

Red blood cells are well-known oxygen and carbon dioxide carriers between the lungs and peripheral tissues.^31^ RBCs are also implicated in the development of atherosclerosis, this occurs when CVD inhibits normal RBC activity due to reduced tissue perfusion in atherosclerosis and hence reduced oxygen supply to peripheral tissue.^31,32^ Our data showed that RBCs, HGB, and HCT levels were significantly lower in STEMI, NSTEMI, UA, HF, DCM, and ICM patients. This is consistent with previous study that demonstrated RBC, HGB, and HCT levels to be much lower in people suffering from acute coronary syndrome (ACS).^23^

Furthermore, a correlation between high RDW and mortality has been demonstrated.^33^ Substantial differences in RDW levels were seen between the HF, DCM, and ICM groups and the control group. Red distribution width is a prognostic indicator for CVD patients, although RDW’s relationship mechanism with cardiovascular diseases remained unclear. The most accepted explanation for the link between RDW and CVDs is that systemic factors include inflammation and oxidative stress play a role. Inflammation appears to produce changes in RBC maturation, anisocytosis, and increased RDW via increasing adrenergic and neuroendocrine activity and stimulating the renin-angiotensin system. Moreover, oxidative stress can raise RDW in acute inflammatory circumstances by damaging RBC membranes and prompting bone marrow (BM) to release immature RBCs into the circulation.^8^

Hemoglobin and RDW parameters were recently introduced as predictors of recurrent hospitalizations; as a consequence, physicians should monitor these levels in diabetic and cancer patients in particular.^34^ As a result, our findings lend credence to the suggested use of various hematological variables as prognostic markers for certain diseases.

According to recent research, platelets play a key role in the pathophysiology of atherothrombosis by having a significant influence on the development of atherosclerotic plaques. Larger, more reactive platelets hasten the formation of an intracoronary thrombus, which leads a variety of heart conditions, including ACS. Unstable angina and myocardial infarction are associated to platelet aggregability. Platelet size and activity are connected, with MPV levels being greater prior to an acute myocardial infarction.^35^ The MPV measures the average size of platelets in the blood as well as the rate at which bone marrow creates them and previous research suggests that increased MPV levels are related to worse cardiac outcomes in ACS patients.^23,35,36^ Another research discovered no connection between higher MPV and death in individuals with coronary artery disease.^24^ In our investigation, we found that the MPV value was considerably lower in the STEMI and UA groups when compared with control, which contradicted prior findings. We propose that the difference between our study and prior studies is that all patients in our study do not have complications and are discharged from the hospital once they have recovered. In contrast, most prior research had focused on the role of high MPV in CVD patients’ increased morbidity and mortality.

Clinical laboratory testing for coagulation, such as the prothrombin time (PT), activated partial thromboplastin time (APTT), and the international normalized ratio (INR), are essential.^37^ We found that the PT, PTT, and INR levels in our investigation were comparable to the findings of prior investigations.^23,24,38-40^ The retrospective study performed by Kırış et al^38^ reported that prolonged initial PT without anticoagulant medication use has been linked to all-cause death in patients with ACS following percutaneous coronary intervention (PCI) and PT can be used to determine ACS patients with high risk. Prothrombin time and APTT were elevated in acute MI patients receiving anticoagulants therapy in a study carried out by Saxena et al^39^ and by Khan et al.^40^ According to the studies carried out by Babes et al^24^ and Adam et al,^23^ PT was useful in predicting mortality and consequences in ACS patients.

Inflammation and coagulation both contribute to the progression of atherosclerosis as well as in the pathogenesis of vascular diseases. Between these 2 systems, there are increasing clues of an interference relationship, which means that one system can trigger the other.^38^ The exposure of tissue factor-bearing inflammatory cells to blood in ruptures of atherosclerotic plaque leads to activation of coagulation.^41^ Furthermore, consumptive coagulopathy can occur because of the elevated inflammation and the activation of the coagulation pathway. As result, many studies have suggested that the prolonged PT in ACS is associated with the inflammation, which contributes to consumptive coagulopathy.^24,38^ However, prolonged PT and INR can occur for other reasons, such as the decreased production of the clotting factor in liver disease or hepatic injury from diminished arterial perfusion or passive congestion in heart failure.^42^

Ischemic heart disease is a group of clinical conditions characterized by myocardial ischemia and a mismatch between blood supply and demand in the myocardium.^43^ Ischemic heart disease is divided into 2 categories: CCS and ACS, with ACS further subdivided into STEMI, NSTEMI, and UA.^44-46^ In our study, we identified 35 patients diagnosed with CCS in this group; we observed a noticeable reduction in some CBC parameters, including RBC, HGB, HCT, and MPV, while coagulation parameters remained unaffected. We discovered a variation in WBC and coagulation markers when we compared these data to an ACS group and previous studies. According to a 2018 study, IHD patients had a considerable rise in WBC count.^47^ Moreover, a higher neutrophil-to-lymphocyte ratio was shown to be significantly linked with overall mortality and cardiovascular disease mortality, but not cancer mortality.^48^

### Study limitation

The key limitation of the research was the inability to have access to data from previous years. Although our retrospective investigation was carried out on available hematological parameters and coagulation profiles between December 2020 and January 2022, larger sample sizes and a longer time of observation may provide a more robust result. In conclusion, even though our data was based on a single source, the Prince Faisal bin Khalid Cardiac Centre, Asir region, they provide an important observation to our study because the center receives patients from all Asir-region cities and is the only cardiac center in Asir-region in Saudi Arabia.

In conclusion, there is a high demand for useful, noninvasive, and widely available prognostic markers that can contribute to disease management. Patients with cardiovascular diseases, in particular, require frequent monitoring to avoid complications that may result in death. The current investigation found considerable aberrant alterations in hematological markers and coagulation profiles in patients with cardiovascular diseases. Thus, routine monitoring of significant markers that could be a sign of disease complications might be utilized for disease management. This study clearly demonstrates a significant frequency of STEMI disease among CVD patients in the Asir region.

Furthermore, coagulation and hematological markers are assumed to have significant diagnostic or prognostic utility. Testing methods are widely accessible and can be utilized to monitor CVD patients, particularly those with ACS or HF. Further research with such a larger sample in different Saudi cities is required to assess the prevalence of CVD and variations in hematological and coagulation parameters between patients at admission and discharge.
